# Probability assessment of intracerebral hemorrhage in prehospital emergency patients

**DOI:** 10.1186/s42466-020-00100-1

**Published:** 2021-01-06

**Authors:** Frederik Geisler, Medschid Wesirow, Martin Ebinger, Alexander Kunz, Michal Rozanski, Carolin Waldschmidt, Joachim E. Weber, Matthias Wendt, Benjamin Winter, Heinrich J. Audebert

**Affiliations:** 1 Department of Neurology, Charité – Universitätsmedizin Berlin, Hindenburgdamm 30, 12203 Berlin, Germany; 2 Department of Neurology, Medical Park Berlin Humboldtmühle, Berlin, Germany; 3 Department of Neurology, Auguste-Viktoria-Klinikum, Berlin, Germany; 4 Department of Neurology, Humboldt-Klinikum, Berlin, Germany; 5 Department of Neurology, Unfallkrankenhaus Berlin, Berlin, Germany; 6 Department of Neurology, St. Josefs-Krankenhaus, Potsdam, Germany; 7 Center for Stroke Research Berlin, Berlin, Germany

**Keywords:** Mobile stroke unit, Intracerebral hemorrhage, Ischemic stroke, Emergency medical services, Vascular neurology

## Abstract

**Background:**

Routing of patients with intracerebral hemorrhage (ICH) and acute ischemic stroke (AIS) to the most appropriate hospital is challenging for emergency medical services particularly when specific treatment options are only provided by specialized hospitals and determination of the exact diagnosis is difficult. We aimed to develop a prehospital score – called prehospital-intracerebral hemorrhage score (ph-ICH score) – to assist in discriminating between both conditions.

**Methods:**

The ph-ICH score was developed with data from patients treated aboard a mobile stroke unit in Berlin, Germany, between 2011 and 2013 (derivation cohort) and in 2018 (validation cohort). Diagnosis of ICH or AIS was established using clinical data and neuroradiological cerebral imaging. Diagnostic accuracy was measured with significance testing, Cohen’s d and receiver-operating-characteristics.

**Results:**

We analyzed 416 patients (32 ICH, 224 AIS, 41 transient ischemic attack, 119 stroke mimic) in the derivation cohort and 285 patients (33 ICH and 252 AIS) in the validation cohort. Systolic blood pressure, level of consciousness and severity of neurological deficits (i. e. certain items of the National Institutes of Health Stroke Scale) were used to calculate the ph-ICH score that showed higher values in the ICH compared to the AIS group (derivation cohort: 1.8 ± 1.2 vs. 1.0 ± 0.9 points; validation cohort: 1.8 ± 0.9 vs. 0.8 ± 0.7 points; d = 0.9 and 1.4, both *p* < 0.01). Receiver-operating-characteristics showed fair and good accuracy with an area under the curve of 0.71 for the derivation and 0.81 for the validation cohort.

**Conclusions:**

The ph-ICH score can assist medical personnel in the field to assess the likelihood of ICH and AIS in emergency patients.

**Supplementary Information:**

The online version contains supplementary material available at 10.1186/s42466-020-00100-1.

## Background

The term stroke derives from the sudden onset of neurological deficits but includes heterogeneous subtypes of acute ischemic stroke (AIS), intracerebral hemorrhage (ICH) and subarachnoid hemorrhage (SAH) [1, 2]. Some therapeutic approaches, such as antithrombotic/thrombolytic treatment, are indicated in AIS patients but are contraindicated in ICH patients. In contrast, acute blood pressure lowering is regularly used in ICH patients to reduce early hematoma growth [3] while such therapy is generally not recommended in AIS patients. Certain clinical features were found to be associated with higher likelihood of ICH and were used to develop clinical decision scores to discriminate between ICH and AIS patients [4], but diagnostic accuracy was rather low [5]. Therefore, ICH can only be reliably diagnosed or excluded by cerebral imaging (computed tomography [CT] or magnetic resonance imaging [MRI]), usually only available in hospitals. Mobile stroke units (MSUs) with imaging capabilities on board offer stroke subtype differentiation in the prehospital setting [6–8].

The use of MSUs has spread in several countries, but they are not yet available in most areas worldwide [9, 10]. Therefore, a prehospital probability estimation of ICH or AIS is based on patient characteristics and clinical examination. Because some time-sensitive interventions like systemic thrombolysis alone or in combination with mechanical thrombectomy [11–13] or neurosurgical operations are only available in specialized hospitals, the differentiation between ICH and AIS patients is clinically relevant to make the correct transport decision to the nearest and most appropriate hospital. Otherwise, secondary transfers from non-specialized hospitals are required, thereby delaying treatment and possibly worsening prognosis.

We aimed at developing and validating a simple clinical decision score, called prehospital-intracerebral hemorrhage (ph-ICH) score, that can be used by paramedics with limited training in neurological examination. Frequently, only limited data are available on previous medical conditions and medication of individual patients in the prehospital setting and usually no prehospital cerebral imaging capabilities are available. Therefore, the ph-ICH score was constructed as a simple prehospital multidimensional score assessing and considering only a few easily obtainable and measurable clinical variables in the absence of cerebral imaging data. This risk stratification ph-ICH score should assist but not replace the prehospital diagnostic steps – depending on certain threshold values – in assessing the probability of ICH and AIS.

## Methods

### Study design

All patients in this study were treated aboard an MSU, called Stroke Emergency Mobile (STEMO) in Berlin, Germany. Further details about STEMO can be found elsewhere [14].

**Fig. 1:**
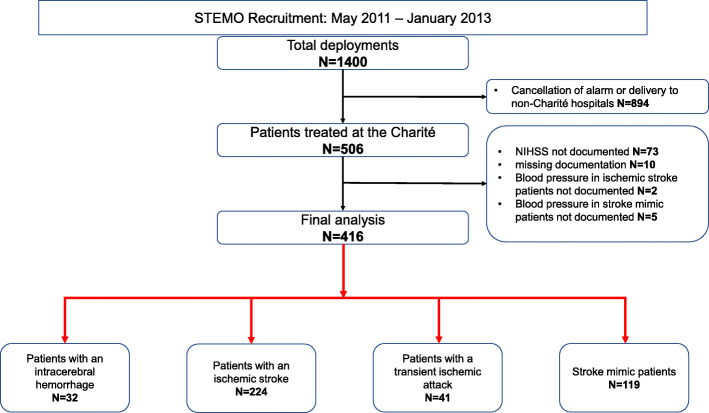
Flow Chart. All patients who were included in the derivation cohort of this study as well as the reasons for exclusion are depicted in the Flow Chart

Patients treated between May 2011 and January 2013 aboard a STEMO that was deployed in the district of Charlottenburg-Wilmersdorf (Ortsteil Wilmersdorf) were analyzed and assigned to a derivation cohort. When STEMO was dispatched, there was a 75% probability of arriving at scene within 16 min and this area covered approximately 1.3 million residents [15, 16]. During this timeframe, the Pre-Hospital Acute Neurological Treatment and Optimization of Medical care in Stroke (PHANTOM-S) study was conducted. This study was approved by the local ethics committee. Details can be found elsewhere [8, 14]. In the derivation cohort patients were classified as ICH, AIS, transient ischemic attack (TIA) or stroke mimic (SM) patients, depending on the final diagnosis in the hospital, as shown in Table 1A. The 1400 patients of the derivation cohort were previously analyzed by our group to distinguish between cerebrovascular disease (CVD) and SM patients [17]. In the derivation cohort patients discharged from one of the three Charité campuses (Campus Benjamin Franklin, Campus Mitte, Campus Virchow Klinikum) with complete documentation were evaluated for further analysis, as shown in the Flow Chart (Fig. 1). We included only patients treated at the Charité, because we did not have access to in-hospital documentation of other hospitals.

**Table 1:** Characteristics of enrolled ICH and AIS patients. The effect size – Cohen’s d – for the derivation and validation cohort as well as the *p*-values for Chi-Square and non-parametric Mann-Whitney-U as well as Fisher’s exact test (not adjusted for multiple testing) are depicted

A) Derivation cohort					
All patients **(*n*** **= 416)** AIS/TIA/ SM **(*n*** **= 384)** TIA patients **(*n*** **= 41)** SM patients **(*n*** **= 119)**	ICH patients **(*n*** **= 32)**	AIS patients **(*n*** **= 224)**	Cohen’s d (pooled SD) AIS-ICH Chi-Square test^#^ Mann-Whitney-U test*		
**Age (years)** [95% CI]	71.5 ± 11.4 [67.3, 75.6]	74.9 ± 12.4 [73.3, 76.6]	0.3 (12.3) *p* = 0.11*		
**No. of female patients** (relative No. in %)	13 (40.6%)	127 (56.7%)	*p* = 0.02^#^		
**SP (mmHg)** (mean average ± SD) [95% CI]	197 ± 34 [185, 210]	164 ± 32 [160, 168]	1.0 (32.5) *p* < 0.01*		
**DP (mmHg)** (mean average ± SD) [95% CI]	110 ± 28 [99, 120]	94 ± 23 [91, 97]	0.7 (23.4) *p* < 0.01*		
**Mean arterial pressure (mmHg)** (mean average ± SD) [95% CI]	139 ± 28 [129, 149]	117 ± 24 [114, 120]	0.9 (24.4) *p* < 0.01*		
**SP ≥ 180 mmHg** (relative No. in %)	21 (65.6%)	72 (32.1%)	*p* < 0.01^#^		
**DP ≥ 110 mmHg** (relative No. in %)	10 (31.3%)	42 (18.8%)	*p* = 0.1^#^		
**MAP ≥ 130 mmHg** (relative No. in %)	17 (53.1%)	55 (24.6%)	*p* < 0.01^#^		
**NIHSS (points)** [median (IQR)]	15 (15)	7 (12)	n. a.		
**NIHSS ≥ 10** No. of patients (in %)	19 (59.4%)	90 (40.2%)	*p* = 0.04^#^		
**NIHSS ≥ 15** No. of patients (in %)	16 (50.0%)	58 (25.9%)	*p* = 0.01^#^		
**NIHSS (LOC)** (mean average ± SD) [95% CI]	0.5 ± 0.9 [0.2, 0.8]	0.2 ± 0.5 [0.2, 0.3]	0.5 (0.6) *p* = 0.11*		
**NIHSS (LOC) ≥ 1** No. of patients (in %)	9 (28.1%)	39 (17.4%)	*p* = 0.15^#^		
**Arterial hypertension** No. of patients (in %)	26 (81.3%)	173 (77.2%)	p = 0.61^#^		
**Atrial fibrillation** No. of patients (in %)	6 (18.8%)	94 (42.0%)	*p* = 0.01^#^		
**Seizure** No. of patients (in %)	0	2 (0.9%)	n. a.		
**ph-ICH score (points) (mean average ± SD)**	**1.8 ± 1.2**	**1.0 ± 0.9**	**0.9 (0.9)** ***p*** < 0.01*		
B) Validation cohort					
All patients **(*n*** **= 285)**	ICH patients **(*n*** **= 33)**	AIS patients **(*n*** **= 252)**		Cohen’s d (pooled SD) AIS-ICH Chi-Square test^#^ Mann-Whitney-U test* Fisher’s exact test^+^	
**Age (years)** [95% CI]	73.3 ± 11.4 [69.2, 77.4]	73.9 ± 13.9 [72.2, 75.7]		0.1 (13.7) *p* = 0.63*	
**No. of female patients** (relative No. in %)	15 (45.5%)	119 (47.2%)		*p* = 0.85^#^	
**SP (mmHg)** (mean average ± SD) [95% CI]	189 ± 35 [176, 202]	163 ± 31 [159, 166]		0.9 (31.2) *p* < 0.01*	
**DP (mmHg)** (mean average ± SD) [95% CI]	104 ± 29 [94, 115]	87 ± 18 [85, 89]		0.9 (19.1) *p* < 0.01*	
**Mean arterial pressure (mmHg)** (mean average ± SD) [95% CI]	133 ± 29 [122, 143]	112 ± 19 [110, 114]		1.0 (20.6) *p* < 0.01*	
**SP ≥ 180 mmHg** (relative No. in %)	20 (60.6%)	83 (32.9%)		*p* < 0.01^#^	
**DP ≥ 110 mmHg** (relative No. in %)	14 (42.4%)	25 (9.9%)		*p* < 0.01^+^	
**MAP ≥ 130 mmHg** (relative No. in %)	16 (48.5%)	45 (17.9%)		*p* < 0.01^#^	
**NIHSS (points)** [median (IQR)]	15 (12)	6 (9)		n. a.	
**NIHSS ≥ 10** No. of patients (in %)	23 (69.7%)	76 (30.2%)		*p* < 0.01^#^	
**NIHSS ≥ 15** No. of patients (in %)	17 (51.5%)	44 (17.5%)		*p* < 0.01^#^	
**NIHSS (LOC)** (mean average ± SD) [95% CI]	0.5 ± 0.8 [0.2, 0.7]	0.1 ± 0.3 [0.1, 0.1]		1.0 (0.4) *p* < 0.01*	
**NIHSS (LOC) ≥ 1** No. of patients (in %)	11 (33.3%)	15 (6.0%)		*p* < 0.01^+^	
**Arterial hypertension** No. of patients (in %)	31 (93.9%)	210 (83.3%)		*p* = 0.11^#^	
**Atrial fibrillation** No. of patients (in %)	5 (15.2%)	79 (31.3%)		*p* = 0.06^#^	
**ph-ICH score (points) (mean average ± SD)**	**1.8 ± 0.9**	**0.8 ± 0.7**		**1.4 (0.7)** ***p*** < 0.01*	
C) ph-ICH score with single items					
**All patients – Derivation cohort** (*n* = 416)	**ICH patients** (*n* = 32)	**AIS/TIA/ SM patients** (*n* = 384)	**AIS patients** (*n* = 224)	**TIA patients** (*n* = 41)	**SM patients** (*n* = 119)
**SP ≥ 180 mmHg** (relative No. in %)	21 (65.6%)	106 (27.6%)	72 (32.1%)	9 (22.0%)	25 (21.0%)
**NIHSS (LOC) ≥ 1** No. of patients (in %)	9 (28.1%)	56 (14.6%)	39 (17.4%)	1 (2.4%)	16 (13.5%)
**NIHSS item: LOC** (mean average ± SD) [95% CI]	0.5 ± 0.9 [0.2, 0.8]	0.2 ± 0.5 [0.1, 0.2]	0.2 ± 0.5 [0.2, 0.3]	0.0 ± 0.3 [0.0, 0.2]	0.2 ± 0.5 [0.1, 0.3]
**NIHSS item: following commands** (mean average ± SD) [95% CI]	0.8 ± 0.9 [0.5, 1.1]	0.4 ± 0.7 [0.4, 0.5]	0.5 ± 0.8 [0.4, 0.6]	0.3 ± 0.7 [0.1, 0.5]	0.4 ± 0.7 [0.3, 0.6]
**NIHSS item: visual field** (mean average ± SD) [95% CI]	0.5 ± 0.8 [0.2, 0.8]	0.2 ± 0.6 [0.2, 0.3]	0.2 ± 0.6 [0.1, 0.3]	0.3 ± 0.8 [0.1, 0.6]	0.2 ± 0.6 [0.1, 0.3]
**NIHSS item: motor weakness (right arm)** (mean average ± SD) [95% CI]	1.8 ± 1.8 [1.1, 2.4]	0.6 ± 1.1 [0.5, 0.7]	0.8 ± 1.3 [0.6, 1.0]	0.1 ± 0.4 [0.0, 0.2]	0.3 ± 0.8 [0.2, 0.5]
**NIHSS item: motor weakness (left arm)** (mean average ± SD) [95% CI]	1.4 ± 1.7 [0.8, 2.0]	0.7 ± 1.3 [0.6, 0.9]	0.9 ± 1.5 [0.7, 1.1]	0.4 ± 1.1 [0.1, 0.8]	0.5 ± 1.1 [0.3, 0.7]
**NIHSS item: motor weakness (right leg)** (mean average ± SD) [95% CI]	1.6 ± 1.7 [1.0, 2.2]	0.6 ± 1.1 [0.5, 0.7]	0.7 ± 1.3 [0.6, 0.9]	0.2 ± 0.6 [0.0, 0.4]	0.4 ± 0.9 [0.3, 0.6]
**NIHSS item: motor weakness (left leg)** (mean average ± SD) [95% CI]	1.2 ± 1.6 [0.7, 1.8]	0.7 ± 1.2 [0.6, 0.8]	0.9 ± 1.4 [0.7, 1.1]	0.3 ± 0.9 [0.1, 0.6]	0.5 ± 1.1 [0.3, 0.7]
**NIHSS item: sensory disturbance** (mean average ± SD) [95% CI]	1.3 ± 0.9 [0.9, 1.6]	0.5 ± 0.8 [0.5, 0.6]	0.7 ± 0.8 [0.6, 0.8]	0.2 ± 0.5 [0.1, 0.4]	0.3 ± 0.6 [0.2, 0.4]
**ph-ICH score**	**1.8 ± 1.2**	**0.8 ± 0.8**	**1.0 ± 0.9**	**0.4 ± 0.6**	**0.6 ± 0.7**
**All patients – Validation cohort** (*n* = 285)	**ICH patients** (*n* = 33)		**AIS patients** (*n* = 252)		
**SP ≥ 180 mmHg** (relative No. in %)	20 (60.6%)		83 (32.9%)		
**NIHSS (LOC) ≥ 1** No. of patients (in %)	11 (33.3%)		15 (6.0%)		
**NIHSS item: LOC** (mean average ± SD) [95% CI]	0.5 ± 0.8 [0.2, 0.7]		0.1 ± 0.3 [0.0, 0.1]		
**NIHSS item: following commands** (mean average ± SD) [95% CI]	0.7 ± 0.8 [0.4, 1.0]		0.3 ± 0.7 [0.2, 0.4]		
**NIHSS item: visual field** (mean average ± SD) [95% CI]	0.2 ± 0.6 [0.0, 0.4]		0.3 ± 0.6 [0.2, 0.3]		
**NIHSS item: motor weakness (right arm)** (mean average ± SD) [95% CI]	1.3 ± 1.7 [0.8, 1.9]		0.6 ± 1.2 [0.5, 0.8]		
**NIHSS item: motor weakness (left arm)** (mean average ± SD) [95% CI]	1.9 ± 1.8 [1.2, 2.5]		0.8 ± 1.3 [0.7, 1.0]		
**NIHSS item: motor weakness (right leg)** (mean average ± SD) [95% CI]	1.4 ± 1.7 [0.8, 2.0]		0.6 ± 1.3 [0.5, 0.8]		
**NIHSS item: motor weakness (left leg)** (mean average ± SD) [95% CI]	1.5 ± 1.7 [0.9, 2.1]		0.8 ± 1.4 [0.6, 1.0]		
**NIHSS item: sensory disturbance** (mean average ± SD) [95% CI]	1.0 ± 0.8 [0.7, 1.3]		0.5 ± 0.7 [0.4, 0.6]		
**ph-ICH score**	**1.8 ± 0.9**		**0.8 ± 0.7**		

*AIS* Acute ischemic stroke, *BP* Blood pressure, *CI* Confidence interval, *DP* Diastolic blood pressure, *ICH* Intracerebral hemorrhage, *IQR* Interquartile range, *LOC* Level of consciousness, *MAP* Mean arterial pressure, *n. a*. Not available, *NIHSS* National Institutes of Health Stroke Scale, *No*. Number, *mmHg* Millimeter of mercury, *ph-ICH score* Prehospital-intracerebral hemorrhage score, *SD* Standard deviation, *SM* Stroke mimic, *SP* Systolic blood pressure, *TIA* Transient ischemic attack

In the validation cohort we evaluated patients treated aboard one of three STEMOs in Berlin, Germany who were registered in the SPecific Acute Treatment in Ischemic or hAemorrhagic Stroke With Long Term Follow-up (B-SPATIAL) database (ClinicalTrials.gov Identifier: NCT03027453) as part of the Berlin PRe-hospital Or Usual Delivery of Acute Stroke Care (B_PROUD) project (ClinicalTrials.gov Identifier: NCT02869386). The three STEMOs that entered data in the B-SPATIAL database were stationed in the districts of Charlottenburg-Wilmersdorf, Tempelhof-Schöneberg and Marzahn-Hellersdorf. In the validation cohort patients were classified as ICH or AIS patients, depending on the final diagnosis in the hospital, as shown in Table 1B.

### Data collection and analysis

Baseline demographics are found in Table 1, the single items of the National Institutes of Health Stroke Scale (NIHSS) in Table 2 and different thresholds for the ph-ICH score in Table 3.

**Table 2:** Single items of the NIHSS in patients with ICH, AIS, TIA and SM (Derivation cohort). The sum score and all items of the NIHSS for the ICH and AIS, TIA, SM patients separately as well as the difference between ICH and AIS/TIA/SM patients are shown. The sum score is depicted as the mean average score for all patients, the units for the single items are shown in points. The items depicted in bold are part of the “short NIHSS” and the ph-ICH score. A pdf version of the NIHSS with an explanation for all items of the score can be found here: https://www.stroke.nih.gov/documents/NIH_Stroke_Scale_508C.pdf

NIHSS (points)	ICH patients (mean average) (***n*** = 32)	AIS/TIA/ SM patients (mean average) (***n*** = 384)	AIS patients (mean average) (***n*** = 224)	TIA patients (mean average) (***n*** = 41)	SM patients (mean average) (***n*** = 119)	Difference (ICH-AIS/TIA/SM) Kruskal-Wallis test
**sum score in points** **(mean average)**	**14.9**	**7.7**	**9.5**	**4.2**	**5.7**	**7.2**
**Level of Consciousness (LOC) (0–3)**	**0.5**	**0.2**	**0.2**	**0.0**	**0.2**	**0.3** ***p*** **= 0.02**
LOC Questions (0–2)	1.0	0.7	0.8	0.5	0.8	0.3 *p* = 0.18
**LOC Commands (0–2)**	**0.8**	**0.4**	**0.5**	**0.3**	**0.4**	**0.4** ***p*** **= 0.02**
Best Gaze (0–2)	0.7	0.3	0.5	0.1	0.2	0.4 *p* < 0.01
**Visual (0–3)**	**0.5**	**0.2**	**0.2**	**0.3**	**0.2**	**0.3** ***p*** **= 0.03**
Facial Palsy (0–3)	1.5	0.9	1.2	0.5	0.6	0.6 *p* < 0.01
**Motor Arm right (0–4)**	**1.8**	**0.6**	**0.8**	**0.1**	**0.3**	**1.2** ***p*** **< 0.01**
**Motor Arm left (0–4)**	**1.4**	**0.7**	**0.9**	**0.4**	**0.5**	**0.6** ***p*** **< 0.01**
**Motor Leg right (0–4)**	**1.6**	**0.6**	**0.7**	**0.2**	**0.4**	**1.0** ***p*** **< 0.01**
**Motor Leg left (0–4)**	**1.2**	**0.7**	**0.9**	**0.3**	**0.5**	**0.5** ***p*** **< 0.01**
Limb Ataxia (0–2)	0.0	0.1	0.1	0.0	0.1	−0.1 *p* = 0.34
**Sensory (0–2)**	**1.3**	**0.5**	**0.7**	**0.2**	**0.3**	**0.7** ***p*** **< 0.01**
Best Language (0–3)	1.2	0.8	0.8	0.6	0.7	0.5 *p* = 0.15
Dysarthria (0–2)	1.1	0.7	0.8	0.4	0.5	0.4 *p* < 0.01
Extinction and Inattention (formerly Neglect) (0–2)	0.4	0.3	0.4	0.1	0.1	0.2 *p* < 0.01

*AIS* Acute ischemic stroke, *ICH* Intracerebral hemorrhage, *LOC* Level of consciousness, *NIHSS* National Institutes of Health Stroke Scale, *SM* Stroke mimic, *TIA* Transient ischemic attack

**Table 3:** A, B and C. Accuracy of the ph-ICH score for the prediction of ICH and AIS/TIA/SM as well as ICH and AIS patients. Table A summarizes the results of AIS/TIA/SM patients and Table B of the AIS patients for the derivation cohort; Table C shows the results of AIS patients for the validation cohort

A) Derivation cohort							
ph-ICH score **SP ≥ 180 + NIHSS LOC ≥ 1 + NIHSS**_**short**_ **(sum of the following NIHSS items: LOC, following commands, visual field, motor weakness of arm or leg, sensory disturbance divided by 10)**						Cohen’s d (pooled SD) **1.2 (0.9)**	
ph-ICH score	**Sensitivity**	**Specificity**	**PPV**	**NPV**	**+LR**	**No. of AIS/TIA/SM patients**	**No. of ICH patients**
≥1.5	0.50	0.80	0.17	0.95	2.5	76 (19.8%)	16 (50.0%)
≥2.0	0.38	0.88	0.21	0.94	3.1	46 (12.0%)	12 (37.5%)
≥2.5	0.28	0.96	0.38	0.94	7.2	15 (3.9%)	9 (28.1%)
≥3.0	0.25	0.97	0.44	0.94	9.6	10 (2.6%)	8 (25.0%)
≥3.5	0.13	1.00	0.80	0.93	48.0	1 (0.3%)	4 (12.5%)
B) Derivation cohort							
ph-ICH score **SP ≥ 180 + NIHSS LOC ≥ 1 + NIHSS**_**short**_ **(sum of the following NIHSS items: LOC, following commands, visual field, motor weakness of arm or leg, sensory disturbance divided by 10)**						Cohen’s d (pooled SD) **0.9 (0.9)**	
ph-ICH score	**Sensitivity**	**Specificity**	**PPV**	**NPV**	**+LR**	**No. of AIS patients**	**No. of ICH patients**
≥1.5	0.50	0.73	0.21	0.91	1.9	60 (26.8%)	16 (50.0%)
≥2.0	0.38	0.83	0.24	0.90	2.3	37 (16.5%)	12 (37.5%)
≥2.5	0.28	0.95	0.45	0.90	5.7	11 (4.9%)	9 (28.1%)
≥3.0	0.25	0.96	0.47	0.90	6.2	9 (4.0%)	8 (25.0%)
≥3.5	0.13	1.00	0.80	0.89	28.0	1 (0.4%)	4 (12.5%)
C) Validation cohort							
ph-ICH score **SP ≥ 180 + NIHSS LOC ≥ 1 + NIHSS**_**short**_ **(sum of the following NIHSS items: LOC, following commands, visual field, motor weakness of arm or leg, sensory disturbance divided by 10)**						Cohen’s d (pooled SD) **1.4 (0.7)**	
ph-ICH score	**Sensitivity**	**Specificity**	**PPV**	**NPV**	**+LR**	**No. of AIS patients**	**No. of ICH patients**
≥1.5	0.52	0.87	0.34	0.93	3.9	33 (13.1%)	17 (51.5%)
≥2.0	0.39	0.94	0.46	0.92	6.6	15 (6.0%)	13 (39.4%)
≥2.5	0.24	0.98	0.62	0.91	12.2	5 (2.0%)	8 (24.2%)
≥3.0	0.12	1.00	1.00	0.90	n. a.	0	4 (12.1%)
≥3.5	0.03	1.00	1.00	0.89	n. a.	0	1 (3.0%)

*AIS* Acute ischemic stroke, *ICH* Intracerebral hemorrhage, *LOC* Level of consciousness, *+LR* Positive likelihood ratio (sensitivity/(1-specificity)), *n. a*. Not available (division by 0), *NIHSS* National Institutes of Health Stroke Scale, *No*. Number, *NPV* Negative predictive value, *ph-ICH score* Prehospital-intracerebral hemorrhage score, *PPV* Positive predictive value, *SD* Standard deviation, *SM* Stroke mimic, *SP* Systolic blood pressure, *TIA* Transient ischemic attack

The STEMO documentation report was used to collect baseline demographics. If baseline information was missing, the discharge letter or emergency department report was used to collect the information. History of arterial hypertension and atrial fibrillation were not always known in the prehospital setting, e. g. due to missing information from relatives and no knowledge about previous illnesses and were taken from the hospital records. Similarly, the presence of a seizure during the prehospital or hospital treatment period were recorded according to hospital documentation. The first measured blood pressure (BP) (systolic blood pressure [SP] and diastolic blood pressure [DP]) and the items of the NIHSS were only gathered from the STEMO documentation. Mean arterial pressure (MAP) was calculated according to the formula: $\frac{\mathrm{SP}}{3}+\left(\frac{2}{3}\right)\times \mathrm{DP}$. In patients with suspected stroke, the NIHSS documentation is mandatory in the STEMO documentation report, but optional for other patients. In cases of missing information patients were excluded from the analysis.

Baseline demographics, statistics, mean averages with their corresponding confidence intervals (CI), the median with the corresponding interquartile range (IQR) were calculated as summarized in Table 1. Furthermore, the absolute and relative number of patients with arterial hypertension, atrial fibrillation and occurrence of seizures are reported. For BP, MAP and NIHSS sum score certain thresholds – as dichotomous variables – are depicted.

### Statistical analysis

We used Chi-Square test for independence with cross-tabulation to test whether two categorial variables from a population were related to each other. In cases of an expected frequency < 5 in one cell of the cross-tabulation, the assumption for Chi-Square test was violated and thereby we used Fisher’s exact test. We additionally measured effect sizes with Cramér’s V (V = 0.1–0.29 small, V = 0.3–0.49 moderate and V ≥ 0.5 large effect). The Mann-Whitney-U test was calculated to detect statistical significant differences for metric variables between independent groups, as shown in Table 1. Tests were two-sided (α = 0.05).

The Kruskal-Wallis test was applied to the single NIHSS items to find possible significant differences between multiple groups and in cases of statistical significance a pairwise comparison with the Dunn-Bonferroni post hoc method compared each group to one another, adjusted for multiple testing with Bonferroni correction.

The corresponding *p*-values for each test are depicted in Tables 1 and 2. Further p-values, Χ^2^ and V are found in the Tables in the Supplement.

We measured effect sizes with Cohen’s d to assess the strength of effects between groups. We additionally used this measure of effect size, because it is – in contrast to null hypothesis significance testing with their corresponding p-values – not affected by sample size and allows to estimate the size of an effect. The following effect sizes for two independent means were proposed by Cohen: d = 0.2 small effect; d = 0.5 medium effect; d = 0.8 large effect [18].

Effect sizes were measured with Cohen’s d to assess the strength of effects between groups. In cases of negative Cohen’s d we used the modulus for easier interpretation throughout this study. Cohen’s d was calculated for the sample according to the formula: $\hat{d}=\frac{\overline{X_1}-\overline{X_2}}{s_P}$. $\overline{X_1}$ and $\overline{X_2}$ representing the mean average values for the ICH and AIS groups for the sample and s_p_ the pooled standard deviation.

Groups were assumed to be independent and pooled standard deviations were calculated according to the formula:

${s}_P=\sqrt{\frac{\left({N}_1-1\right){s}_1^2+\left({N}_2-1\right){s}_2^2}{N_1+{N}_2-2}}$. N_1_ and N_2_ representing sample size, s_1_ and s_2_ the standard deviation for each sample.

For the differentiation between ICH and AIS (as well as TIA/SM) diagnosis, the ph-ICH score was developed as a clinical decision rule. The derivation is described in more detail in the Discussion. The score was calculated as the sum of one point for SP ≥180 mmHg, one point for level of consciousness ≥1 and the sum of certain single items of the NIHSS divided by ten (level of consciousness, following commands, visual field, motor weakness of an arm or a leg and sensory disturbance), as shown in Table 3. The sum of all single NIHSS items by ten minimizes the impact of the neurological deficit to the score. The individual items of the ph-ICH score are depicted in Table 2C.

The validity (i. e. sensitivity, specificity, positive and negative predictive values (PPV and NPV)) and the positive likelihood ratio (+LR) (sensitivity/1-specificity) for differentiating between ICH and AIS/TIA/SM as well as between ICH and AIS patients (Table 3) were calculated. Certain threshold values for the ph-ICH score can be found in Table 3 for the derivation and validation cohort. The +LR values of ≥3 and ≥ 10 were interpreted as moderate and strong likelihood of one condition over the other.

**Fig. 2:**
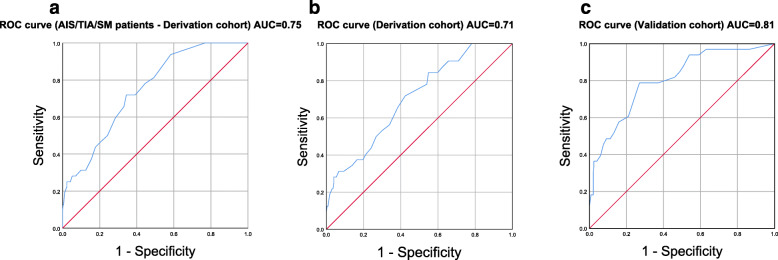
ROC curve. ROC curve with the AUC for the derivation and validation cohort. **a** shows the AIS/TIA/SM group for the derivation cohort, **b** the AIS group for the derivation cohort and C the AIS group for the validation cohort. AIS: acute ischemic stroke; AUC: area under the curve; ICH: intracerebral hemorrhage; ROC: receiver-operating-characteristics; SM: stroke mimic

A receiver-operating-characteristics (ROC) curve analysis with an area under the curve (AUC) for the ph-ICH score was performed to assess the accuracy (Fig. 2). The ph-ICH score was used as the test variable and the diagnosis (AIS/TIA/SM)/ICH and AIS/ICH as the state variable (value of the state variable = ICH). An AUC of 0.50–0.59 indicates a fail, 0.60–0.69 poor, 0.70–0.79 fair, 0.80–0.89 good and 0.90–1.0 excellent accuracy of a diagnostic test in model prediction.

IBM SPSS 25 (IBM, Armonk, New York, United States) and Microsoft Excel 2016 (Microsoft, Redmond, United States) were used for calculations and statistical tests.

## Results

A total of 1400 STEMO alarms were evaluated, and 416 patients were identified with complete documentation in the derivation cohort, as shown in the Flow Chart (Fig. 1). AIS was diagnosed in 224 (53.9%), SM in 119 (28.6%), TIA in 41 (9.9%) and ICH in 32 (7.7%) patients. For the validation cohort, we analyzed data of 252 AIS (88.4%) and 33 ICH (11.6%) patients (285 patients overall). The baseline demographics can be found in Table 1 and Table 1 of the Supplement.

No significant age differences were found. In the derivation cohort ICH patients were more likely male (*p* = 0.02).

The ph-ICH score showed significant higher mean average scores for ICH compared to the AIS group (derivation cohort: 1.8 ± 1.2 vs. 1.0 ± 0.9, d = 0.9, *p* < 0.01; validation cohort: 1.8 ± 0.9 vs. 0.8 ± 0.7 points, d = 1.4, *p* < 0.01). The ph-ICH scores were lower in the SM (derivation cohort: 0.6 ± 0.7 points) and TIA group (derivation cohort: 0.4 ± 0.6 points).

The specificity, PPV and LR+ were positively and the sensitivity was negatively correlated with increasing ph-ICH scores in the AIS group, as indicated in Table 3B and C. Increasing ph-ICH scores increased the likelihood that a patient suffers from an ICH and not an AIS. When evaluating certain threshold values, ph-ICH scores of greater than 1.5, 2.0, 2.5, 3.0 and 3.5 showed a likelihood for an ICH, i. e. a PPV of 0.21, 0.24, 0.45, 0.47, 0.8 and 0.34, 0.46, 0.62, 1.00, 1.00 for the derivation and validation cohort (Table 3B and C), respectively.

The ICH patients presented with higher first measured SP and DP compared to the group of AIS patients in the derivation cohort (mean average: 197 ± 34/110 ± 28 vs. 164 ± 32/94 ± 23 mmHg, d = 1.0 and 0.7, both *p* < 0.01) and validation cohort (mean average: 189 ± 35/104 ± 29 vs. 163 ± 31/87 ± 18 mmHg, both d = 0.9 and *p* < 0.01). Accordingly, a higher relative number of patients showed a SP ≥ 180 mmHg and DP ≥ 110 mmHg (derivation cohort: 65.6 vs. 32.1% and 31.3 vs. 18.8%, *p* < 0.01 and *p* = 0.1; validation cohort: 60.6 vs. 32.9% and 42.4 vs. 9.9%, both *p* < 0.01).

Stroke severity was higher in the ICH group compared to the AIS group (derivation cohort: median NIHSS sum score: 15 (Q_1_-Q_3_ = 7–22, IQR = 15) vs. 7 (Q_1_-Q_3_ = 4–16, IQR = 12); validation cohort: median NIHSS sum score: 15 (Q_1_-Q_3_ = 7–19, IQR = 12) vs. 6 (Q_1_-Q_3_ = 3–12, IQR = 9)). Accordingly, a higher relative number of ICH patients showed an NIHSS sum score ≥ 10 (derivation cohort: 59.4 vs. 40.2%, *p* = 0.04; validation cohort: 69.7 vs. 30.2%, *p* < 0.01) and ≥ 15 (derivation cohort: 50.0 vs. 25.9%, *p* = 0.01; validation cohort: 51.5 vs. 17.5%, *p* < 0.01) compared to AIS patients. SM patients presented with lower NIHSS sum scores than AIS patients and TIA patients presented with the lowest NIHSS sum scores. More ICH patients showed a decrease in level of consciousness.

Similar proportions of patients with a history of arterial hypertension were found in the ICH and AIS group (derivation cohort: 81.3 vs. 77.2%, *p* = 0.61; validation cohort: 93.9 vs. 83.3%, *p* = 0.11) while atrial fibrillation was found less often in the derivation cohort for the ICH group (derivation cohort: 18.8 vs. 42.0%, *p* = 0.01). Overall, 25 seizures were reported in the SM and two in the AIS group.

## Discussion

ICH and AIS patients require very different, frequently highly time-critical, medical interventions, often only available in certain specialized hospitals. Therefore, clinical prediction scores were developed to assess the likelihood of an ICH and AIS based on clinical judgement [19]. The Siriraj Stroke Score – based on eight items and tested in small studies with a limited number of patients – seems to lack positive predictive value for both ICH and AIS patients [20, 21]. Other authors report a higher validity for decision scores, but require prerequisites usually not available in prehospital care like the neurological assessment of the patient after 3 hours and paraclinical variables (white blood cell count) [22]. Other authors conclude, that the Siriraj and Guy’s hospital stroke score [5] and the Allen score [23] also lack accuracy in distinguishing ICH from AIS patients.

Here, we developed a prehospital decision score, called ph-ICH score, to assess the likelihood of ICH or AIS patients with certain requirements: a) the score is easy to calculate with only a limited number of variables, b) can be performed without extensive neurological knowledge and c) does not require information about pre-existing conditions of the patient.

SP ≥ 180 mmHg and the level of consciousness≥1 (one point for each item) as two dichotomous variables can be easily determined and were different between ICH and AIS patients and were therefore included in the ph-ICH score. The single NIHSS item level of consciousness was particularly investigated, because a reduced level of consciousness is often reported to be more likely in ICH than AIS patients [4].

Furthermore, for reasons of simplicity, we chose the single items of the NIHSS that most likely can be performed by non-neurological specialists and showed significant differences between ICH and AIS patients. We developed this “short NIHSS” comprising vigilance, following commands, visual field, motor weakness of an arm or leg as well as sensory disturbances with data from the derivation cohort und validated this score within the ph-ICH score in the validation cohort. The “short NIHSS” variables showed significant differences between ICH and AIS patients and may be assessed by non-neurological personnel without extensive training in neurological examination. The ph-ICH score was calculated as the sum of the dichotomous variables SP ≥ 180 mmHg, level of consciousness≥1 (1 point for each item) and the sum of the “short NIHSS”. SP and level of consciousness were used as dichotomous variables for reasons of simplicity. To adjust the “short NIHSS” to the level of blood pressure and level of consciousness, the sum was divided by ten, as shown in Table 3. We did not include atrial fibrillation in the ph-ICH score, because it requires information about pre-existing conditions which may not always be available in the prehospital setting. However, if available in the field, the presence of atrial fibrillation may additionally be used to assess the likelihood of ICH or AIS.

The likelihood of suffering from an ICH rises with increasing ph-ICH scores (Table 3). Because the paramedics do not know for certain whether the patient with a suspected CVD suffers from an ICH, AIS, TIA or SM, we additionally compared the ICH with the AIS/TIA/SM group. In this comparison, when choosing certain threshold scores of greater than 1.5 and especially 3.0, the likelihood of an ICH steeply rises with increasing values, as reflected in the PPV and positive likelihood ratio.

Although the PPV, likelihood ratio and relative number of patients for ICH is very high above certain threshold values (≥3.0), the low prevalence of an ICH is resulting in a similar absolute number of patients (9 vs. 8 patients, Table 3B), because the PPV depends on the prevalence of a disease.

Similar results were found when comparing the differences of the ph-ICH score between the ICH and AIS group as well as the ICH and AIS/TIA/SM group (Table 3).

The ROC curve performed fair and good with an AUC of 0.71 and 0.81 (Fig. 2).

In addition to the above mentioned variables, a number of clinical findings have been reported to increase the likelihood of an ICH compared to an AIS diagnosis such as coma, neck stiffness, seizures accompanying the neurologic deficit, DP > 110 mmHg, vomiting and headache [4]. On average, patients with ICH present with more severe neurological deficits [24].

These results are in line with our findings of higher SP and DP, higher proportion of patients with an impaired consciousness and more severe neurological deficits, i. e. higher median NIHSS sum scores. Because neck stiffness, vomiting and headache were not documented in a standardized manner, these variables were not investigated in this study.

Seizures are often mimicking a CVD and in a study of our group 21% of SM patients had seizures [17]. Most seizures in this study were also found in the SM group (25 patients, 92.6%).

Certain limitations must be considered. First, our analysis was conducted retrospectively on already existing study data with no monitoring, possibly leading to some data abstraction inaccuracies. Second, the ph-ICH score was not actually applied by the emergency medical personnel in the field, but retrospectively calculated and tested. Third, in the validation cohort we were only able to compare ICH to AIS patients. Fourth, the number of AIS patients was considerably larger than the number of ICH patients. The possibility that the results of the ICH groups were found by chance was larger than in the AIS groups. Furthermore, the PPV is dependent on the prevalence of a disease. Fifth, the data of our study was obtained in a highly standardized manner by specialized personnel with extensive experience in the treatment of patients with CVD. Although the ph-ICH-score was developed as a simple tool for paramedics, the generalizability of our results in settings with non-specialized personnel needs to be examined in further studies.

## Conclusions

In summary, ICH compared to AIS patients presented with higher ph-ICH scores and thereby with more severe strokes and higher first measured blood pressures. TIA and SM patients presented with even lower ph-ICH scores and first measured blood pressures than AIS patients. Especially very high values of at least 3.0 and 3.5, increase the likelihood of an ICH over an AIS. The differentiation between ICH and AIS is important, because these patients often require highly time-critical interventions, only available in certain hospitals.

Future larger prospective studies are necessary to investigate whether the ph-ICH score helps to improve the transport decision by emergency medical personnel and thereby improve the outcome of patients.

## Supplementary Information

**Additional file 1.**

## Data Availability

All data analyzed during this study are included in this published article.

## Abbreviations

AFIB: Atrial fibrillation
AIS: Acute ischemic stroke
AUC: Area under the curve
B_PROUD: Berlin PRe-hospital Or Usual Delivery of Acute Stroke Care
B-SPATIAL: Specific Acute Treatment in Ischemic or haemorrhagic Stroke With Long Term Follow-up database
BP: Blood pressure
CI: Confidence interval
CT: Computed tomography
CVD: Cerebrovascular disease
DP: Diastolic blood pressure
ICH: Intracerebral hemorrhage
IQR: Interquartile range
+LR: Positive likelihood ratio
LOC: Level of consciousness
MAP: Mean arterial pressure
MRI: Magnetic resonance imaging
MSU: Mobile stroke unit
n. a.: Not available
NIHSS: National Institutes of Health Stroke Scale
No.: Number
mmHg: Millimeter of mercury
NPV: Negative predictive value
ph-ICH score: Prehospital-intracerebral hemorrhage score
PHANTOM-S: Pre-Hospital Acute Neurological Treatment and Optimization of Medical care in Stroke study
PPV: Positive predictive value
ROC: Receiver-operating-characteristics
SAH: Subarachnoid hemorrhage
SD: Standard deviation
SM: Stroke mimic
SP: Systolic blood pressure
STEMO: Stroke Emergency Mobile
TIA: Transient ischemic attack
