# Supplementary material for: Probability assessment of intracerebral hemorrhage in prehospital emergency patients
Source: Neurol Res Pract. 2021 Jan 6;3:1. doi: 10.1186/s42466-020-00100-1 (PMC7786495; doi:10.1186/s42466-020-00100-1)
Supplement: Supplementary file 1 — Additional file 1. [file 42466_2020_100_MOESM1_ESM.docx]

***Study Setting***

Stroke Emergency Mobile (STEMO) was alarmed by the central dispatch center of the Berlin Fire Brigade when an acute stroke with a time of onset ≤ 4 hours or unknown was suspected. STEMO was equipped with a Computed Tomography (CT)-scanner and point-of-care laboratory devices and staffed by a vascular neurologist who was also trained in emergency medicine, a paramedic (“Notfallsanitäter” or “Rettungsassistent”) and a radiology technician (“medizinisch-technischer Radiologieassistent”). During STEMO weeks, STEMO and an additional regular ambulance (“Rettungswagen”) were simultaneously deployed. The paramedics on regular ambulances were able to cancel STEMO before its arrival based on their first assessment.

After arrival at scene, the STEMO neurologist assessed and managed possible life-threatening conditions and - if an acute stroke was suspected (even as a possible differential diagnosis) – performed a neurological examination and documented the results on the basis of the National Institutes of Health Stroke Scale (NIHSS). Certain laboratory results, like point-of-care blood glucose, international normalized ratio and other variables (e. g. thrombocytes) were measured when indicated by the neurologist. If systemic thrombolysis or other acute therapeutic consequences required cerebral imaging, the neuroradiologist on call together with the neurologist at scene decided to perform a CT-scan with or without additional CT-angiography. The neuroradiologist on call examined and assessed the imaging data that were sent via teleradiology and informed the STEMO neurologist via telephone about his findings. In some difficult cases, a senior neurologist was involved via telephone, especially in cases when intravenous thrombolysis was considered. After completion of the most important preclinical interventions, the patient was transported to the nearest hospital deemed to be most appropriate for the individual patient, e. g. in cases of large vessel occlusion to a center with a neuroradiology department offering endovascular treatment.

***Study protocol***

Three sources of documentation were used in the derivation cohort for analysis: a) the STEMO documentation report, b) the discharge letter (in cases of patient admission) and c) the emergency department report. The classification and assignment to the intracerebral hemorrhage (ICH), acute ischemic stroke (AIS), transient ischemic attack (TIA) or stroke mimic (SM) group, depended on the final diagnosis stated in the physician’s letter or the emergency department report, in cases patients were not admitted to a ward, because in these cases no discharge letter was available. The final diagnosis was never based on the STEMO documentation report that included the diagnosis of the STEMO neurologist at scene. In the absence of cerebral imaging paramedics in the field usually do not know whether the patient will later be diagnosed with an ICH, AIS, TIA or SM. Therefore, in the derivation cohort we also included TIA and SM patients to evaluate whether the prehospital-intracerebral hemorrhage (ph-ICH) score can distinguish between ICH and TIA and SM patients. We did not have access to this information in the validation cohort.

The definition of ICH always required radiological evidence in CT or magnetic resonance imaging (MRI). Only patients with parenchymal hemorrhage were included. Patients with epidural, subdural hematoma or subarachnoid hemorrhage were excluded from further analysis. They often present with other symptoms than ICH or AIS patients, like a sudden onset of strong headache or a slower evolvement of neurological deficits. The definition of AIS was based on an acute neurological dysfunction of ischemic vascular origin within the brain with a duration longer than 24 hours or any duration if evidence of brain infarction in CT or MRI was found, relevant to the deficit [2].

In cases of diagnostic uncertainty and thereby especially in AIS or TIA patients with minor neurological deficits (admitted to a ward or discharged from the emergency department) with no evidence of brain infarction in cerebral imaging, the physician had the option to note a definitive diagnosis, a most likely diagnosis and in some cases additionally a less likely differential diagnoses. In cases with no definitive diagnoses, the diagnosis described as the most likely diagnosis was used.

Certain differences in the validation cohort compared to the derivation cohort are described hereafter. First, no discharge or emergency department letters were used for further analysis, but the B-SPATIAL database. However, the final diagnosis stated in this database was usually based on the diagnosis from a physician`s letter from a hospital. We did not have access to these letters. The STEMO documentation was used for first prehospital measured and documented blood pressure results and NIHSS single items. Second, patients between January 2018 and October 2018 with complete documentation were included. Patients with missing documentation or unclear diagnoses were excluded. Third, no information about the occurrence of seizures was available. Fourth, only ICH and AIS patients were included in the analysis.

**Table 1.** Characteristics of enrolled ICH, AIS, TIA and SM patients and the effect size – Cohen’s d – for the derivation and validation cohort as well as the p-values for Chi-Square and non-parametric Mann-Whitney-U as well as Fisher’s exact test (not adjusted for multiple testing) are depicted.

A) Derivation cohort

| All patients  **(n=416)** | ICH patients  **(n=32)** | AIS/TIA/ SM patients  **(n=384)** | AIS patients  **(n=224)** | TIA patients  **(n=41)** | SM patients  **(n=119)** | Cohen’s d (pooled SD)  Chi-Square test^#^  Mann-Whitney-U test*  AIS-ICH  TIA-ICH  SM-ICH  AIS/TIA/SM-ICH |
| --- | --- | --- | --- | --- | --- | --- |
| **Age (years)** [95% CI] | 71.5±11.4  [67.3, 75.6] | 73.6±13.9  [72.2, 75.0] | 74.9±12.4  [73.3, 76.6] | 77.1±14.0  [72.6, 81.6] | 69.8±15.5  [66.9, 72.6] | 0.3 (12.3)  p=0.11*  0.4 (12.9)  0.1 (14.8)  0.2 (13.7) |
| **No. of female patients**  (relative No. in %) | 13 (40.6%) | 210 (54.7%) | 127 (56.7%) | 19 (46.3%) | 64 (46.2%) | p=0.02^#^  Χ^2^=5.3  Cramér’s V=0.1 |
| **SP (mmHg)**  (mean average±SD) [95% CI] | 197±34  [185, 210] | 159±34  [156, 163] | 164±32  [160, 168] | 156±31  [146, 166] | 151±37  [145, 157.8] | 1. (32.5)   p<0.01*  1.3 (32.3)  1.3 (36.0)  1.1 (34.1) |
| **DP (mmHg)**  (mean average±SD) [95% CI] | 110±28  [99, 120] | 92±22  [90, 94] | 94±23  [91, 97] | 94±21  [87, 101] | 88±22  [85, 92] | 0.7 (23.4)  p<0.01*  0.7 (24.4)  0.9 (23.0)  0.8 (22.8) |
| **Mean arterial pressure (mmHg)**  (mean average±SD) [95% CI] | 139±28  [129, 149] | 115±24  [112, 117] | 117±24  [114, 120] | 115±23  [107, 122] | 109±25  [105, 114] | 0.9 (24.4)  p<0.01*  1.0 (25.3)  1.1 (25.9)  1.0 (24.8) |
| **SP ≥180 mmHg**  (relative No. in %) | 21 (65.6%) | 106 (27.6%) | 72 (32.1%) | 9 (22.0%) | 25 (21.0%) | p<0.01^#^  Χ^2^=13.6  Cramér’s V=0.2 |
| **DP ≥110 mmHg**  (relative No. in %) | 10 (31.3%) | 71 (18.5%) | 42 (18.8%) | 10 (24.4%) | 19 (16.0%) | p=0.1^#^  Χ^2^=2.7  Cramér’s V=0.1 |
| **MAP ≥130 mmHg**  (relative No. in %) | 17 (53.1%) | 92 (24.0%) | 55 (24.6%) | 11 (26.8%) | 26 (21.9%) | p<0.01^#^  Χ^2^=11.3  Cramér’s V=0.2 |
| **NIHSS (points)**  (mean average±SD) [95% CI] | 14.9±8.9  [11.6, 18.2] | 7.7±7.0  [7.0, 8.4] | 9.5±7.3  [8.5, 10.4] | 4.2±5.4  [2.5, 5.9] | 5.7±5.9  [4.6, 6.7] | 0.7 (7.5)  p<0.01*  1.5 (7.1)  1.4 (6.6)  1.1 (7.2) |
| **NIHSS (points)**  [median (IQR)] | 15 (15) | 6 (10) | 7 (12) | 2 (6) | 4 (7) | n. a. |
| **NIHSS ≥10**  No. of patients (in %) | 19 (59.4%) | 122 (31.8%) | 90 (40.2%) | 8 (19.5%) | 24 (20.2%) | p=0.04^#^  Χ^2^=4.2  Cramér’s V=0.1 |
| **NIHSS ≥15**  No. of patients (in %) | 16 (50.0%) | 72 (18.8%) | 58 (25.9%) | 3 (7.3%) | 11 (9.2%) | p=0.01^#^  Χ^2^=7.9  Cramér’s V=0.2 |
| **NIHSS (LOC)**  (mean average±SD) [95% CI] | 0.5±0.9  [0.2, 0.8] | 0.2±0.5  [0.1, 0.2] | 0.2±0.5  [0.2, 0.3] | 0.0±0.3  [0.0, 0.1] | 0.2±0.5  [0.1, 0.3] | 0.5 (0.6)  p=0.11*  0.7 (0.7)  0.5 (0.6)  0.6 (0.6) |
| **NIHSS (LOC) ≥1**  No. of patients (in %) | 9 (28.1%) | 56 (14.6%) | 39 (17.4%) | 1 (2.4%) | 16 (13.5%) | p=0.15^#^  Χ^2^=2.1  Cramér’s V=0.1 |
| **Arterial hypertension**  No. of patients (in %) | 26 (81.3%) | 272 (70.8%) | 173 (77.2%) | 36 (87.8%) | 63 (53.0%) | p=0.61^#^  Χ^2^=0.3  Cramér’s V=0.0 |
| **Atrial fibrillation**  No. of patients (in %) | 6 (18.8%) | 132 (34.4%) | 94 (42.0%) | 13 (31.7%) | 25 (21.0%) | p=0.01^#^  Χ^2^=6.3  Cramér’s V=0.2 |
| **Seizure**  No. of patients (in %) | 0 | 27 (7.0%) | 2 (0.9%) | 0 | 25 (21.0%) | n. a. |
| **ph-ICH score (points)**  (mean average±SD) | 1.8±1.2 | 0.8±0.8 | 1.0±0.9 | 0.4±0.6 | 0.6±0.7 | 0.9 (0.9)  p<0.01*  1.5 (0.9)  1.4 (0.9)  1.2 (0.9) |

B) Validation cohort

| All patients  **(n=285)** | ICH patients  **(n=33)** | AIS patients  **(n=252)** | Cohen’s d  (pooled SD)  Chi-Square test^#^  Mann-Whitney-U test*  Fisher’s exact test^+^  Cramér’s V |
| --- | --- | --- | --- |
| **Age (years)** [95% CI] | 73.3±11.4  [69.2, 77.4] | 73.9±13.9  [72.2, 75.7] | 0.1 (13.7)  p=0.63* |
| **No. of female patients**  (relative No. in %) | 15 (45.5%) | 119 (47.2%) | p=0.85^#^  Χ^2^=0.0  V=0.0 |
| **SP (mm Hg)**  (mean average±SD) [95% CI] | 189±35  [176, 202] | 163±31  [159, 166] | 0.9 (31.2)  p<0.01* |
| **DP (mm Hg)**  (mean average±SD) [95% CI] | 104±29  [94, 115] | 87±18  [85, 89] | 0.9 (19.1)  p<0.01* |
| **Mean arterial pressure (mmHg)**  (mean average±SD) [95% CI] | 133±29  [122, 143] | 112±19  [110, 114] | 1.0 (20.6)  p<0.01* |
| **SP ≥180 mmHg**  (relative No. in %) | 20 (60.6%) | 83 (32.9%) | p<0.01^#^  Χ^2^=9.7  V=0.2 |
| **DP ≥110 mmHg**  (relative No. in %) | 14 (42.4%) | 25 (9.9%) | p<0.01^+^  V=0.3 |
| **MAP ≥130 mmHg**  (relative No. in %) | 16 (48.5%) | 45 (17.9%) | p<0.01^#^  Χ^2^=16.3  V=0.2 |
| **NIHSS (points)**  (mean average±SD) [95% CI] | 14.5±8.1  [11.6, 17.4] | 7.6±6.0  [6.9, 8.4] | 1.1 (6.3)  p<0.01* |
| **NIHSS (points)**  [median (IQR)] | 15 (12) | 6 (9) | n. a. |
| **NIHSS ≥10**  No. of patients (in %) | 23 (69.7%) | 76 (30.2%) | p<0.01^#^  Χ^2^=20.1  V=0.3 |
| **NIHSS ≥15**  No. of patients (in %) | 17 (51.5%) | 44 (17.5%) | p<0.01^#^  Χ^2^=20.1  V=0.3 |
| **NIHSS (LOC)**  (mean average±SD) [95% CI] | 0.5±0.8  [0.2, 0.7] | 0.1±0.3  [0.1, 0.1] | 1.0 (0.4)  p<0.01* |
| **NIHSS (LOC) ≥1**  No. of patients (in %) | 11 (33.3%) | 15 (6.0%) | p<0.01^+^  V=0.3 |
| **Arterial hypertension**  No. of patients (in %) | 31 (93.9%) | 210 (83.3%) | p=0.11^#^  Χ^2^=2.5  V=0.1 |
| **Atrial fibrillation**  No. of patients (in %) | 5 (15.2%) | 79 (31.3%) | p=0.06^#^  Χ^2^=3.7  V=0.1 |
| **ph-ICH score (points)**  (mean average±SD) | 1.8±0.9 | 0.8±0.7 | 1.4 (0.7)  **p<0.01*** |

Table 2

**Table 2.** Single items of the NIHSS in patients with ICH, AIS, TIA and SM (Derivation cohort)

In addition to Table 2 in the main text, p-values after a pairwise comparison for each group with one another with the Dunn-Bonferroni method – corrected for multiple testing with the Bonferroni method – are shown. The Dunn-Bonferroni method was only used in cases of statistical significance (p<0.05) after applying the Kruskal-Wallis test to a single NIHSS item.

| NIHSS | ICH - AIS patients | ICH - TIA patients | ICH - SM patients | AIS - TIA patients | AIS – SM patients | TIA - SM |
| --- | --- | --- | --- | --- | --- | --- |
| **sum score in points**  **(mean average)** | **14.9** | **7.7** | **9.5** | **4.2** | **5.7** | **7.2** |
| **Level of Consciousness (LOC) (0-3)** | **p=0.52** | **p=0.01** | **p=0.20** | **p=0.11** | **p=1.00** | **p=0.61** |
| LOC Questions (0-2) | **n. a.** | **n. a.** | **n. a.** | **n. a.** | **n. a.** | **n. a.** |
| **LOC Commands (0-2)** | **p=0.09** | **p=0.01** | **p=0.08** | **p=0.82** | **p=1.00** | **p=1.00** |
| Best Gaze (0-2) | **p=0.15** | **p<0.01** | **p<0.01** | **p=0.01** | **p<0.01** | **p=1.00** |
| **Visual (0-3)** | **p=0.08** | **p=0.66** | **p=0.02** | **p=1.00** | **p=1.00** | **p=1.00** |
| Facial Palsy (0-3) | **p=0.38** | **p<0.01** | **p<0.01** | **p<0.01** | **p<0.01** | **p=1.00** |
| **Motor Arm right (0-4)** | **p=0.01** | **p<0.01** | **p<0.01** | **p<0.01** | **p=0.01** | **p=0.61** |
| **Motor Arm left (0-4)** | **p=0.65** | **p=0.01** | **p=0.01** | **p=0.08** | **p=0.02** | **p=1.00** |
| **Motor Leg right (0-4)** | **p=0.01** | **p<0.01** | **p<0.01** | **p=0.03** | **p=0.45** | **p=0.81** |
| **Motor Leg left (0-4)** | **p=1.00** | **p=0.03** | **p=0.05** | **p=0.05** | **p=0.04** | **p=1.00** |
| Limb Ataxia (0-2) | **n. a.** | **n. a.** | **n. a.** | **n. a.** | **n. a.** | **n. a.** |
| Sensory (0-2) | **p=0.01** | **p<0.01** | **p<0.01** | **p<0.01** | **p<0.01** | **p=1.00** |
| Best Language (0-3) | **n. a.** | **n. a.** | **n. a.** | **n. a.** | **n. a.** | **n. a.** |
| Dysarthria (0-2) | **p=0.59** | **p<0.01** | **p<0.01** | **p<0.01** | **p<0.01** | **p=1.00** |
| Extinction and Inattention  (formerly Neglect) (0-2) | **p=1.00** | **p=0.25** | **p=0.09** | **p=0.10** | **p<0.01** | **p=1.00** |
